# The influence of curricula and role models on medical students’ professional self-identification: a reminder call

**DOI:** 10.1007/s40037-015-0203-y

**Published:** 2015-07-10

**Authors:** Ahmed Abu-Zaid, Lynn Alkhatib, Syeda Mina

**Affiliations:** College of Medicine, Alfaisal University, PO Box 50927, 11533 Riyadh, Saudi Arabia

Ravindran and colleagues [1] published an attention-grabbing *Letter* highlighting a noteworthy, yet forgotten, subject entitled: *The issue of* ‘*informed consent*’ *in medical student introductions*. The authors emphasized the continuing improper self-identification attitude by clerkship students, and the associated negative impacts on patient autonomy and the student/doctor-patient relationship. Moreover, they proposed a plausible approach on how students should properly introduce themselves to obtain patients’ informed consent.

However, the authors did not shed light on the importance of medical school curricula and the vital roles played by clerkship directors and attendings as valuable means to rectify the above-mentioned neglected attitude.

Medical schools bear the principal responsibility for educating clerkship students about the standards of professionalism governing clerkship education at teaching hospitals, particularly, the proper self-identification attitude as ‘*medical students*’. Formal comprehensive early integration of this core element of professionalism into medical curricula is highly advised. How best such learning objectives should be delivered and integrated into medical curricula is an issue to be primarily determined by the Curriculum Committee at each medical school. Additionally, in line with the universal move towards student-centred education, inputs from pre-clerkship and clerkship students regarding this subject offer valuable contributions in designing the medical curricula and should be highly considered.

The power of clerkship directors and attendings as educators, mentors and role models cannot be underestimated in influencing students’ attitudes. Unfortunately, sometimes, there is a huge discrepancy between what is learned in formal courses and what is actually conveyed at the bedside and in corridors—the hidden curriculum [2]. For instance, many attendings are often careless about notifying patients about the fact that they are interacting with clerkship students during student-performed educational patient encounters (history-taking, physical examination, counselling) despite institutional regulations regarding obtaining informed consent [3]. The message to students may be that proper self-identification is neither required nor valued. Truly informed consent may be threatened in the process. Indeed, to highlight the importance of self-identification, medical students’ professional introductions should be appropriately valued in all clerkship assessment strategies such as objective structured clinical examinations (OSCEs).

Little is known about whether patient-related factors (e.g., age, gender, nationality/ethnicity, socioeconomic class, highest educational level) influence students’ professional introductions. This potentially productive area of research has barely been explored in the literature. Consequently, medical educators will be able to recognize the influence of these factors, and subsequently identify corrective educational measures to be fittingly addressed in the medical curricula.

Lastly, we would like to commend the authors and felicitate the editors for drawing our attention into this limitlessly essential medical education topic for further discussion.

## Source(s) of support in the form of grants

None.

## Conflicts of interests

None.

## References

[1] Ravindran RP, Lester DG, Nawab KA, Vivekanantham S (2015). The issue of ‘informed consent’ in medical student introductions. Perspect Med Educ.

[2] Stern DT (1998). Practicing what we preach? An analysis of the curriculum of values in medical education. Am J Med.

[3] Cohen DL, McCullough LB, Kessel RW, Apostolides AY, Alden ER, Heiderich KJ (1987). Informed consent policies governing medical students’ interactions with patients. J Med Educ.

